# Exogenous Applications of Brassinosteroids Improve Color of Red Table Grape (*Vitis vinifera* L. Cv. “Redglobe”) Berries

**DOI:** 10.3389/fpls.2018.00363

**Published:** 2018-04-06

**Authors:** Alexis E. Vergara, Katy Díaz, Rodrigo Carvajal, Luis Espinoza, José A. Alcalde, Alonso G. Pérez-Donoso

**Affiliations:** ^1^Departamento de Fruticultura y Enología, Facultad de Agronomía e Ingeniería Forestal, Pontificia Universidad Católica de Chile, Santiago, Chile; ^2^Departamento de Química, Universidad Técnica Federico Santa María, Valparaíso, Chile

**Keywords:** anthocyanins, berry color, brassinosteroids, color index for red grapes (CIRG), quality

## Abstract

Color and other quality parameters of “Redglobe” grape (*Vitis vinifera* L.) berries were evaluated after treatment with brassinosteroid (BR) analogs. Three BRs analogs (24-epibrassinolide, Triol, or Lactone) were applied at three concentrations (0.0, 0.4, or 0.8 mg⋅L^-1^), at the onset of veraison. A commercial formulation (B-2000^®^) was also applied, at a recommended rate of 0.06 mg⋅L^-1^. The tested BR analogs were effective improving berry color (evaluated as color index for red grapes, CIRG), increasing the levels of soluble solids and anthocyanins, and changing the types of anthocyanins present without altering other quality and yield parameters. The effects of BR analogs on color enhancement could be explained by an increase in soluble solids content and/or anthocyanin content. Treatment with 24-epibrassinolide (at 0.4 mg⋅L^-1^) or the commercial formulation tended to favor the production of dihydroxylated anthocyanins, which are responsible for the red and pink colors of grape berries. Results indicate that the use of BRs constitutes a potential tool in the production of table grapes. This is the first report of this enhancement effect in a productive context.

## Introduction

The assessment of table grape quality is traditionally based on sensory attributes such as flavor, aroma, texture, and color. Numerous studies together with demographic observations have demonstrated the importance of color as the main quality indicator on which consumer acceptance of fruit is based (Clydesdale, 1993).

Often, minimum quality standards can be obtained by performing good management practices in the vineyard (Cameron, 1994; Schrader et al., 1994), but in the case of grape varieties such as “Redglobe,” which has large-sized berries and clusters, these practices can easily lead to overproduction. Under these conditions, “Redglobe” berries fail to develop suitable red color (Kliewer and Weaver, 1971; Schrader et al., 1994; Kliewer and Dokoozlian, 2005). In addition, the production of table grapes in Chile is conducted in areas with warm climatic and/or microclimatic conditions. These high temperature conditions inhibit the accumulation of anthocyanins (Mori et al., 2005), negatively affecting the development of berry color (Spayd et al., 2002; Mori et al., 2005; Kuhn et al., 2014). Thus, a series of techniques that tend to correct and/or prevent these problems have been implemented. These techniques include the use of growth regulators such as ethephon and/or abscisic acid (ABA) during veraison; these techniques have been widely used on varieties such as “Flame Seedless” and “Redglobe.” However, softening problems have been reported in “Redglobe” berries as a consequence of ethephon and ABA applications, especially at higher doses. In addition, the results of such treatments are not consistent between growing seasons, and these treatments can lead to the expression of darker colors in berries, which renders “Redglobe” berries less attractive in their target markets (Peppi et al., 2006, 2007; Peppi and Fidelibus, 2008). The side effects associated with the use of ethephon and/or ABA can be reduced by the use of lower doses of these compounds; however, those lower doses are also less effective at increasing the anthocyanin content (Peppi et al., 2007) and berry color.

From this point of view, the alternatives to growth regulators for preventing and/or correcting color development problems appear to be limited. Therefore, new growth regulators must be identified and characterized for inclusion in management programs. In this sense, brassinosteroids (BRs) are associated with the ripening of berries (Symons et al., 2006), and exogenous application of 24-epibrassinolide (a BR analog) increases the accumulation of phenolic compounds (Luan et al., 2013; Xi et al., 2013) such as anthocyanins, which are secondary metabolites that determine the color of the berries.

Brassinosteroids represent a group of hormones first isolated from pollen extracts of *Brassica napus* L. (Mitchell et al., 1970). The isolation of brassinolide, the most active of these hormones (Grove et al., 1979), and the identification of its receptor (Wang et al., 2001) made it possible to study this hormone in various species, including grape species. Symons et al. (2006) reported that BRs are involved in the ripening of “Cabernet Sauvignon” berries. Recent studies have shown that BRs are involved in the accumulation of sugar during the ripening of “Cabernet Sauvignon” (Xu et al., 2015). On the other hand, exogenous applications of 24-epibrassinolide during veraison are effective at increasing sugar accumulations, reducing total acidity at harvest and significantly increasing the total anthocyanin content in “Cabernet Sauvignon” (Luan et al., 2013; Xi et al., 2013). Although the observed increase in total anthocyanins may be associated with an increase in the color of grape berries, this phenomenon has not been proven in any table grape variety, especially red ones, for which berry color is one of the main attributes of quality (Clydesdale, 1993). In addition, all studies related to the ripening of berries and to the effect of BRs on ripening have used only one analog (24-epibrassinolide), limiting the understanding and scope that growth regulators analogous to BRs may have within a productive context. Therefore, the objective of the present work was to evaluate the effects of the exogenous applications of different BR analogs on the development and final quality of “Redglobe” berries.

## Materials and Methods

### Plant Material and Experimental Conditions

The experiments were carried out during 2014–2015 and 2015–2016 growing seasons in a commercial vineyard located in Santa María (Aconcagua Province, Valparaíso Region, Chile; 32°43′00.9"S 70°37′56.7"W). The climate corresponding to the study site is Mediterranean, which consists of a dry season during the summer (from December to February).

Sixteen-year-old self-rooted “Redglobe” grapevines (*Vitis vinifera* L.) of similar vigor, health, and fruit load (38 ± 1.1 clusters⋅plant^-1^) were used in the study. The vines were spaced 3.5 m within rows and 3.5 m between rows, and the rows were oriented in southwest–northeastern direction. The vines were trained on an overhead trellis (parronal español) with four main arms and were pruned on a spur-cane system; each cane contained six to seven buds. In both seasons plants were under commercial management and subjected to plant growth regulator application program showing in **Table 1**. However, the plants used in the experiments did not receive the application of ethephon, thus avoiding its interference on the development of fruit color.

**Table 1:** Detail of plant growth regulator application program in the commercial field in which the study was conducted.

Date	Objective	Commercial product	Active ingredient (ai)	Dose (g⋅ha^-1^)
06-10-14	Shoot elongation	Splendor^®^	Thidiazuron, 5.0% (p/v)	2.055
11-11-14	Thinning	Proggib^®^	Gibberellic acid, 3.2% (p/v)	1.8563
22-11-14	Berry growth^∗^	Splendor^®^	Thidiazuron, 5.0% (p/v)	0.4
27-11-14	Berry growth^∗^	Proggib^®^	Gibberellic acid, 3.2% (p/v)	37.4998
		Biozime TF^®^	Gibberellic acid, 0.036% (p/v)	0.0539
			Indoleacetic acid, 0.036% (p/v)	0.0539
			Zeatin, 0.0094% (p/v)	0.1409
02-12-14	Berry growth^∗^	Proggib^®^	Gibberellic acid, 3.2% (p/v)	37.4998
22-01-15	Color uniformity^∗∗^	Ethrel 48^®^	Ethephon, 48.0% (p/v)	239.928

*^∗^Growth regulator applications were performed with a total volume of 1000 L⋅ha^–1^. ^∗∗^Not applied to the plants used in this study.*

### Treatments

**FIGURE 1:**
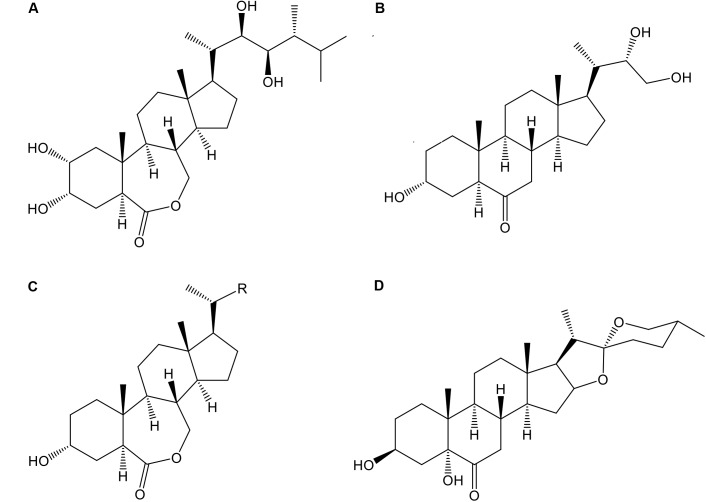
Chemical structure of the different BR analogs used in this study: 24-epibrassinolide **(A)**, 3α-22(*S*), 23-trihydroxy-24-nor-5α-cholan-6-one (**B**, Triol), 3α-hydroxy-20-R-B-homo-7-oxa-5α-cholestan-6-one (**C**, Lactone), and 3β-5α-25(R)-dihydroxy-spirostan-6-one (**D**, commercial formulation, B-2000^®^).

In the season 2014–2015 three analogs of BRs, 24-epibrassinolide (E; Phyto Technology Laboratories^®^, United States), 3α-22(*S*), 23-trihydroxy-24-nor-5α-cholan-6-one (Triol; T), and 3α-hydroxy-20-R-B-homo-7-oxa-5α-cholestan-6-one (Lactone; L) at concentrations of 0.0 (control), 0.4, and 0.8 mg⋅L^-1^ as well as a commercial formulation of BRs, B-2000^®^ (B-2000; IONA, Chile), at concentration of 0.06 mg⋅L^-1^ (equivalent to 60 mg⋅ha^-1^ using 1000 liters of solution per ha, i.e., dose recommended by manufacturer) were applied with a backpack sprayer to “Redglobe” clusters during berry softening at the beginning of veraison (January 7, 2015) at a rate of approximately 1.2 L⋅plant^-1^ until runoff, ensuring that all berry surfaces were covered. The details of the treatments are shown in **Table 2**, and the chemical structures of the analogs are shown in **Figure 1**. The side chain of the structure of the analog Lactone is not shown, as it may be patented and/or protected by copyright; hence, the side chain was replaced with an R.

**Table 2:** Details of the treatments applied in growing season 2014–2015 and/or 2015–2016.

Treatment	Active ingredient	Concentration (mg·L^–1^)
E-0.4^∗^	24-epibrassinolide	0.4
E-0.8		0.8
T-0.4^∗^	3α-22(*S*), 23-trihydroxy-24-nor-5α-cholan-6-one	0.4
T-0.8	(Triol)	0.8
L-0.4^∗^	3α-hydroxy-20-R-B-homo-7-oxa-5α-cholestan-6-one	0.4
L-0.8	(Lactone)	0.8
B-2000^∗^	(25R)-3β-5α-dihydroxy-spirostan-6-one	0.06
	(B-2000)	
Control^∗^		0.0

*E-0.4, 24-epibrassinolide applied at 0.4 mg·L^–1^; E-0.8, 24-epibrassinolide applied at 0.8 mg·L^–1^; T-0.4, Triol applied at 0.4 mg·L^–1^; T-0.8, Triol applied at 0.8 mg·L^–1^; L-0.4, lactone applied at 0.4 mg·L^–1^; L-0.8, lactone applied at 0.8 mg·L^–1^; B-2000, B-2000 applied at 0.06 mg·L^–1^; Control, control treatment. The treatments were applied to cluster at the beginning of veraison at a rate close to 1.2 L·plant^–1^. ^∗^Treatments evaluated in a second season (2015–2016).*

The solutions of BR analogs were prepared by dissolving 4 or 8 mg in 100 mL of 98% ethanol (v/v) and bringing each solution to a final volume of 10 L with water. The control solution was prepared adding water to 100 mL of 98% ethanol (v/v) until reaching 10 L, and the commercial formulation solution was prepared as specified by the manufacturer. A wetting agent (Break^®^, BASF, Germany) was added at a rate of 0.2 mL⋅L^-1^ to all solutions.

Additionally, and using the same methodology described above, at the beginning of veraison of 2015–2016 growing season (January 28, 2016), the same analogs were tested, but only at 0.4 mg⋅L^-1^. The commercial formulation at 0.06 mg⋅L^-1^ was also tested.

### Preharvest and Harvest Evaluations

The evolution of berry skin color, equatorial diameter, and soluble solids were measured using 30 berries per plant; berries were sampled from at least 10 clusters. Sampling was performed at 20-day intervals after treatment application until harvest [57 and 61 days after veraison (dav) for 2014–2015 and 2015–2016 seasons, respectively].

Berry skin color was evaluated using the color index for red grapes (CIRG), which is based on the CIELAB parameters *L*^∗^ (lightness), *H* (hue angle), and *C* (chroma) (Carreño et al., 1995) measured with a Chroma Meter CR-400 (Konica Minolta, Japan) and is calculated as (180 – *H*)/(*L*^∗^+*C*). The CIRG exhibits strong linearity with the visual color of berries and can distinguish between sample groups of different external color (Carreño et al., 1995). The CIRG was measured on 8-mm-diameter circles (50.26 mm^2^) on two opposite sides of the equatorial zone of berries. For this evaluation, all berries were cleaned with a cotton cloth to remove dust and prevent irregularities in the measurements. Berry diameter at the equatorial zone was measured with a digital caliper. Finally, the juice of 10 berries was used for determining the soluble solids concentration (°Brix), using a temperature-compensated hand refractometer.

At harvest, four clusters per plant were sampled. Fifty berries were collected from each cluster and were used for the evaluation previously described (30 for berry skin color, diameter, and soluble solids); in addition, total acidity (expressed as tartaric acid), total anthocyanins, and the profiles of anthocyanin groups were evaluated using 10 whole berries pert plant.

Total acidity was measured using the mixed juice of 10 berries; juice was titrated with NaOH (0.1 N) until pH 8.2 was reached using an automatic titrator (HI 901 Tiratatro, Hanna Instrument, United States).

The total concentration [mg⋅g^-1^ fresh weight (FW)] and total content (mg⋅berry^-1^) of anthocyanins were evaluated using a spectrophotometric method described by Iland et al. (2004). Briefly, 10 whole berries were ground and macerated with 50% ethanol (v/v) at pH 2.0 for 24 h in darkness at room temperature. Afterward, the samples were centrifuged. The supernatants were recovered, and their absorbance at 520 nm was measured with a UV–visible spectrophotometer (Nanodrop 2000, Thermo Fisher, United States) to determine total anthocyanin concentrations (Iland et al., 2004).

To determine the profiles of the different groups of anthocyanins, the extracts obtained in the previous step were subjected to HPLC analysis in accordance with the methodology described by González et al. (2012). HPLC-diode-array detection (DAD) analysis was performed using a LaChrom Elite^®^ HPLC system with a 1.024 photodiode-array detector (Hitachi LaChrom Elite, Japan). Separation was performed using a Purospher^®^ STAR (Merck, Germany) reverse-phase C18 column (250 mm, 4.6 mm i.d., 5 μm) at 25°C; the detection was carried out at 520 nm. The elution gradient consisted of two solvents: solvent A consisted of 90:10 water:formic acid (v/v) and solvent B consisted of acetonitrile (Fanzone et al., 2010). Aliquots of 200 μL of grape extract were injected after being filtered through a 0.45-μm-pore size membrane. To determine the groups of anthocyanins [dihydroxylated (2-OH), trihydroxylated (3-OH), methylated (Met), and non-methylated (Non-met) groups], standard solutions of delphinidin-3-glucoside, petunidin-3-glucoside, malvidin-3-glucoside, cyanidin-3-glucoside, and peonidin-3-glucoside (Extrasynthese, France) were used as standards.

### Experimental Design and Statistical Analysis

A randomized complete block design was used for both growing seasons. Treatments were sorted in five blocks within two rows of plants; this design left one untreated plant as a border between adjacent experimental units (a single plant with all its clusters). Differences among treatment means of preharvest and harvest parameters were evaluated by the analysis of variance (ANOVA), and significant differences were subjected to the Tukey–Kramer honestly significant difference (HSD) multiple comparison test (*p* ≤ 0.05). In addition, correlation analysis were performed to determine the correlation strength between the different evaluated parameters.

## Results

### Quality Parameters

**FIGURE 2:**
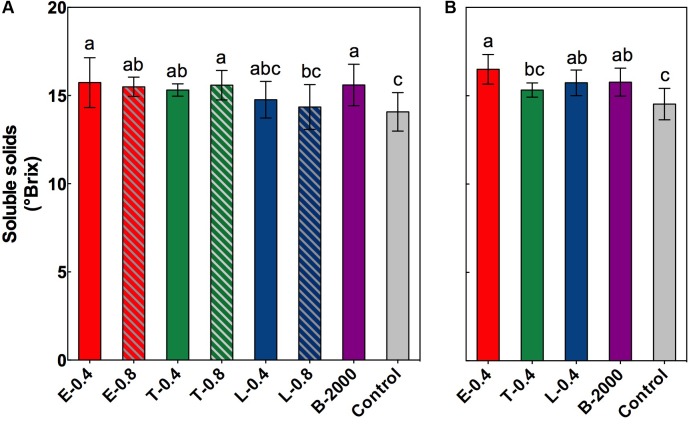
Soluble solids content, expressed as °Brix, at harvest for the growing seasons 2014–2015 **(A)** and 2015–2016 **(B)**. Each bar indicates the mean of five replicates and error lines correspond to standard deviations. Different letters indicate significant differences at *p* < 0.05 according to the Tukey–Kramer HSD test (*n* = 5).

At harvest, in both seasons, no significant differences were observed in equatorial diameter, total acidity, or weight of the berries or clusters (Supplementary Tables 1, 2). However, significant differences were found in the soluble solids content of berries (**Figures 2A,B**). On average, compared with the control treatment, BR applications led to higher soluble solids content in berries. In the season 2014–2015 the soluble solids content of berries that received treatments E-0.4, B-2000, T-0.8, E-0.8, and T-0.4 were significantly different from that of the control berries, whereas treatments L-0.4 and L-0.8 did not significantly differ respect to control treatment in this parameter (**Figure 2A**). These results were consistent with those observed in the 2015–2016 season, in which the treatments E-0.4 and B-2000 presented values statistically higher than those of control treatment.

### Color of Berries

**FIGURE 3:**
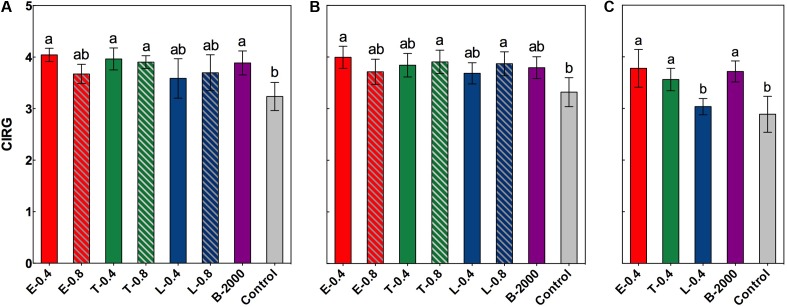
Effect of BRs treatments on CIRG at harvest for the growing seasons 2014–2015 **(A)**, BRs effect in growing season 2014–2015 considering the soluble solids effect **(B)**, and BRs effect on CIRG at harvest in growing season 2015–2016 **(C)**. Each bar indicates the mean of five replicates and error lines correspond to standard deviations. Different letters indicate significant differences at *p* < 0.05 according to the Tukey–Kramer HSD test (*n* = 5).

In both seasons an increase in the values of CIRG was observed from the moment of the application of the treatments until the harvest (data not shown). However, significant differences in CIRG values were observed only at harvest (**Figures 3A–C**).

In the season 2014–2015, ANOVA results and subsequent multiple comparison analyses showed that treatments E-0.4, T-0.4, T-0.8, and B-2000 resulted in significantly higher CIRG values than the control treatment, whereas the remaining treatments resulted in CIRG values that were similar to those of the control treatment (**Figure 3A**). However, analysis of covariance indicated a significant effect of soluble solids content on CIRG value. Nevertheless, and considering the effect of soluble solids content, treatments E-0.4, T-0.8, and L-0.8 still had a statistically significant effect on berry color (**Figure 3B**). On the other hand, the interaction between treatment and soluble solids content with respect to the CIRG was not significant, indicating that effects of treatments on CIRG value did not vary with the level of soluble solids. Although no significant effect of soluble solids content was observed on the CIRG values in the 2015–2016 season, a significant increase in the values of CIRG was observed in treatments E-0.4, T-0.4, and B- 2000 with respect to those of control treatment (**Figure 3C**).

### Total Anthocyanins and Anthocyanin Groups

**FIGURE 4:**
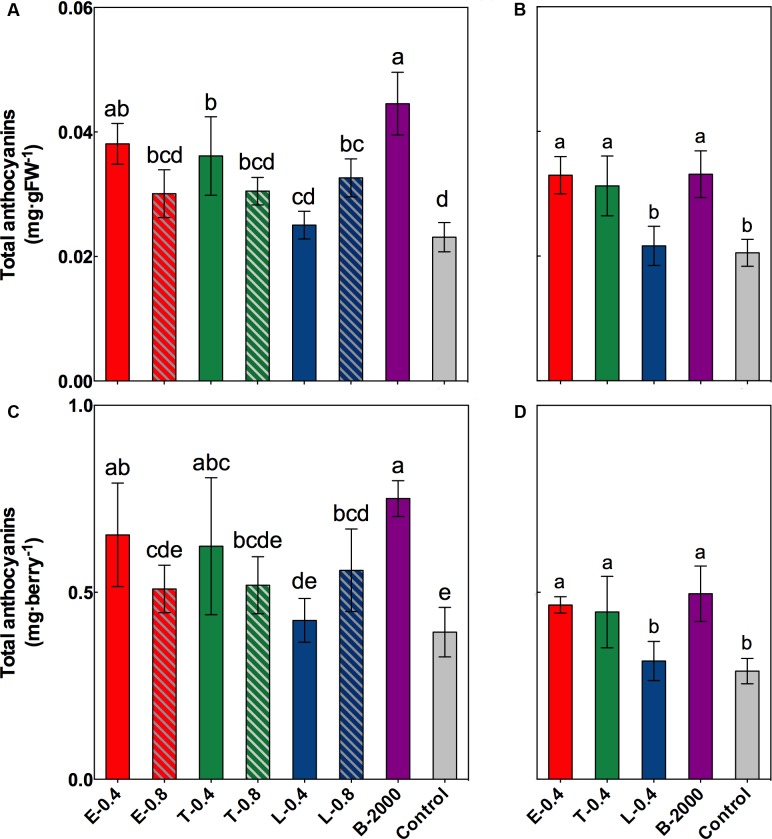
Effect of BR treatments on the total anthocyanin content at harvest, expressed as milligram per gram of fresh weight **(A,B)** or milligram per berry **(C,D)**, for the growing seasons 2014–2015 **(A,C)** and 2015–2016 **(B,D)**. The bars indicate means of five replicates and error lines show their standard deviation. Different letters indicate significant differences at *p* < 0.05 according to the Tukey–Kramer HSD test (*n* = 5).

The results of the statistical analysis of total concentration and content of anthocyanins at harvest revealed that in season 2014–2015 the anthocyanin in grapes that received treatments B-2000, E-0.4, T-0.4, and L-0.8 were significantly higher than those in grapes that received the control treatment; although the other treatments (E-0.8, T-0.8, and L-0.4) resulted in average values that were higher than those of the control, these differences were not statistically significant (**Figures 4A,C**). Despite the fact that in 2015–2016 season the concentration and content of anthocyanins were lower than in the 2014–2015 season (**Figure 4**), a significant increase in the concentration and anthocyanin content was observed in the treatments E-0.4, T-0.4, and B-2000 with respect to the control treatment (**Figures 4B,D**), which is consistent with the results of the previous season.

The observed changes in total anthocyanin concentration and content were also reflected in the concentration and content of specific groups of anthocyanins (**Tables 3**, **4**). The predominant anthocyanins in all of the samples of both seasons were 2-OH anthocyanins (cyanidin-3-glucoside and peonidin-3-glucoside) rather than 3-OH anthocyanins (delphinidin-3-glucoside, petunidin-3-glucoside, and malvidin-3-glucoside). In the case of 2014–2015 season, ANOVA results revealed that all treatments yielded 2-OH anthocyanins levels that were significantly higher than those of 3-OH anthocyanins, whereas no significant difference in the levels of 2-OH and 3-OH anthocyanins was observed in the control berries (**Table 3**). Compared with the control treatment, treatments B-2000, E-0.4, T-0.4, L-0.8, and T-0.8 resulted in a higher content of 2-OH anthocyanins in berries, whereas the level of 2-OH anthocyanins in berries that received the L-0.4 treatment was the same as in berries that received the control treatment. With respect to 3-OH anthocyanins, only treatments T-0.4, B-2000, and T-0.8 resulted in levels that were significantly higher than those from the control treatment (**Table 3**). On the other hand, in the 2015–2016 season, treatments E-0.4, T-0.4, and B-2000 exhibited statistically higher values of 2-OH anthocyanins than the control treatment; whereas L-0.4 treatment remained not different from the control treatment (**Table 4**).

**Table 3:** Effects of BR treatments on the abundance of dihydroxylated, trihydroxylated, methylated, and non-methylated anthocyanins as well as the dihydroxylated:trihydroxylated anthocyanin and the methylated:non-methylated anthocyanin ratios for season 2014–2015.

Treatments	Anthocyanins (mg⋅berry^-1^)				2-OH⋅3-OH^-1^	Met⋅Non-met^-1^
	2-OH	3-OH	Met	Non-met		
E-0.4	0.439 ± 0.1209^∗^ ab	0.214 ± 0.0296 abc	0.451 ± 0.1037^∗^ ab	0.203 ± 0.0359 b	2.052 ± 0.4687 a	2.205 ± 0.1677 a
E-0.8	0.299 ± 0.0547^∗^ cde	0.210 ± 0.0204 abc	0.319 ± 0.0396^∗^ cde	0.190 ± 0.0284 b	1.426 ± 0.2572 b	1.698 ± 0.1799 c
T-0.4	0.378 ± 0.0933^∗^ bc	0.245 ± 0.0948 ab	0.416 ± 0.1101^∗^ abc	0.208 ± 0.0761 b	1.597 ± 0.2381 ab	2.083 ± 0.3377 ab
T-0.8	0.292 ± 0.0456^∗^ cde	0.227 ± 0.0337 ab	0.310 ± 0.0377^∗^ de	0.209 ± 0.0392 ab	1.294 ± 0.1259 b	1.496 ± 0.1143 c
L-0.4	0.243 ± 0.0475^∗^ de	0.182 ± 0.0148 bc	0.267 ± 0.0474^∗^ de	0.159 ± 0.0143 bc	1.331 ± 0.1940 b	1.679 ± 0.2036 c
L-0.8	0.333 ± 0.0702^∗^ bcd	0.226 ± 0.0410 ab	0.362 ± 0.0656^∗^ bcd	0.197 ± 0.0450 b	1.471 ± 0.0913 ab	1.861 ± 0.0949 abc
B-2000	0.507 ± 0.0413^∗^ a	0.243 ± 0.0218 a	0.481 ± 0.0379^∗^ a	0.270 ± 0.0123 a	2.097 ± 0.2376 a	1.783 ± 0.0933 bc
Control	0.215 ± 0.0381 e	0.179 ± 0.0346 c	0.253 ± 0.0433^∗^ e	0.140 ± 0.0258 c	1.217 ± 0.1963 b	1.815 ± 0.1813 bc

*2-OH, dihydroxylated anthocyanins; 3-OH, trihydroxylated anthocyanins; Met, methylated anthocyanins; Non-met, non-methylated anthocyanins. Each value represents the mean of five replicates. Different letters indicate significant differences among treatments for the same type of anthocyanins at p < 0.05 according to the Tukey–Kramer HSD test (n = 5). Asterisks indicate significant differences within the same treatment for different groups of anthocyanins (2-OH vs. 3-OH or Met vs. Non-met) at p < 0.05 according to the Tukey–Kramer HSD test (n = 5).*

**Table 4:** Effects of BR treatments on the abundance of dihydroxylated, trihydroxylated, methylated, and non-methylated anthocyanins as well as the dihydroxylated:trihydroxylated anthocyanin and the methylated:non-methylated anthocyanin ratios for season 2015–2016.

Treatments	Anthocyanins (mg⋅berry^-1^)				2-OH⋅3-OH^-1^	Met⋅Non-met^-1^
	2-OH	3-OH	Met	Non-met		
E-0.4	0.280 ± 0.0250^∗^ a	0.101 ± 0.0120 abc	0.286 ± 0.0268^∗^ ab	0.095 ± 0.0208 a	2.829 ± 0.5982 a	3.169 ± 0.8671 ab
T-0.4	0.236 ± 0.0450^∗^ a	0.112 ± 0.0208 ab	0.257 ± 0.0306^∗^ b	0.091 ± 0.0333 a	2.139 ± 0.3646 ab	3.099 ± 0.9255 ab
L-0.4	0.145 ± 0.0275^∗^ b	0.090 ± 0.0056 bc	0.168 ± 0.0202^∗^ c	0.067 ± 0.0132 a	1.616 ± 0.2933 b	2.570 ± 0.3718 b
B-2000	0.293 ± 0.0578^∗^ a	0.121 ± 0.0122 a	0.325 ± 0.0442^∗^ a	0.088 ± 0.0240 a	2.413 ± 0.3481 a	3.802 ± 0.6181 a
Control	0.131 ± 0.0340^∗^ b	0.084 ± 0.0145 c	0.154 ± 0.0265^∗^ c	0.065 ± 0.0113 a	1.631 ± 0.4067 b	2.440 ± 0.6471 b

*2-OH, dihydroxylated anthocyanins; 3-OH, trihydroxylated anthocyanins; Met, methylated anthocyanins; Non-met, non-methylated anthocyanins. Each value represents the mean of five replicates. Different letters indicate significant differences among treatments for the same type of anthocyanins at p < 0.05 according to the Tukey–Kramer HSD test (n = 5). Asterisks indicate significant differences within the same treatment for different groups of anthocyanins (2-OH vs. 3-OH or Met vs. Non-met) at p < 0.05 according to the Tukey-Kramer HSD test (n = 5).*

In both seasons, the analysis of the 2-OH:3-OH anthocyanin ratio (2-OH⋅3-OH^-1^) indicated that only treatments B-2000 and E-0.4 yielded a ratio that was significantly greater than that found in control berries (**Tables 3**, **4**).

Compared with the control treatment, in 2014–2015 season, treatments B-2000, E-0.4, T-0.4, and L-0.8 resulted in the highest levels of Met anthocyanins (peonidin-3-glucoside, petunidin-3-glucoside, and malvidin-3-glucoside); the levels resulting from the remaining treatments did not significantly differ from those from the control treatment (**Table 3**). These results were consistent with those observed in the 2015–2016 season (**Table 4**).

**FIGURE 5:**
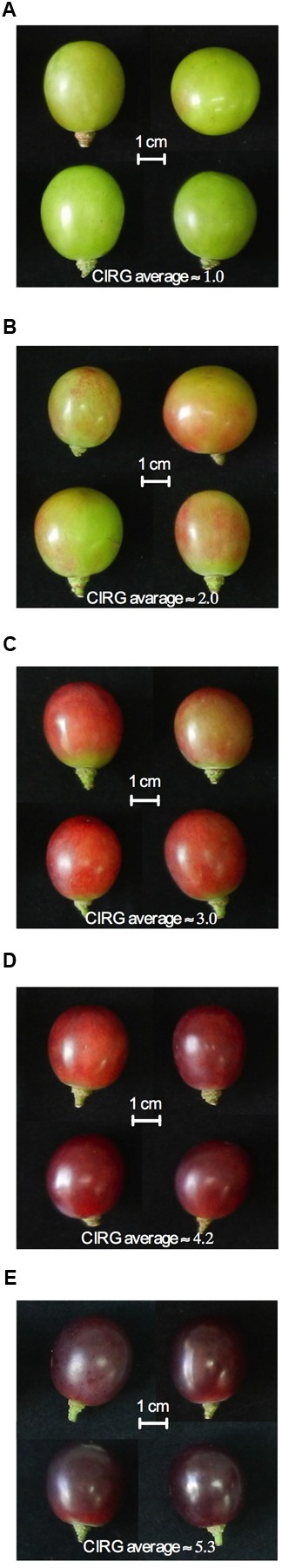
Visual appearance of berries with different mean CIRG values from 1.0 to 5.3 **(A–E)**. In each image, the average CIRG value from the four berries shown is indicated.

In the case of Non-met anthocyanins (cyanidin-3-glucoside and delphinidin-3-glucoside), in season 2014–2015, all treatments, with the exception of L-0.4, produced significantly higher values than the control treatment (**Table 3**). In relation to the ratio between these two groups of anthocyanins, only E-0.4 significantly increased compared to the control treatment, while the rest of treatments remained not different from the control treatment (**Table 3**). While in 2015–2016 season, no significant differences were observed in the content of Non-met anthocyanins, and only the B-2000 treatment presented ratio values of these two groups statistically higher than the control (**Table 4**). Correlation analyzes revealed that berry color (expressed as CIRG) observed in this work (**Figure 5**) are explained by the content and concentration (**Table 5**) of total anthocyanins. On the other hand, the correlation between the content and concentration of the different groups of anthocyanins and CIRG turned out to be significant, especially for the 2-OH anthocyanins expressed as mg⋅berry^-1^ (**Table 5**), while the correlation between CIRG and anthocyanins 3-OH was significant only when these were expressed as mg⋅g^-1^ (**Table 5**).

**Table 5:** Details of correlation between the color of the berries (expressed as the CIRG) and the different groups of anthocyanins and the total anthocyanins, expressed as mg⋅berry^-1^ or mg⋅g FW^-1^.

Anthocyanins	mg⋅berry^-1^		mg⋅g FW^-1^	
	*p*	*R*	*p*	*R*
2-OH	0.0062^∗^	0.4252	0.0002^∗^	0.5555
3-OH	0.3009	0.1676	0.0079^∗^	0.4138
Met	0.0151^∗^	0.3814	0.0002^∗^	0.5533
Non-met	0.0317^∗^	0.3401	0.0007^∗^	0.5131
Total anthocyanins	0.0153^∗^	0.3804	0.0001^∗^	0.5659

*2-OH, dihydroxylated anthocyanins; 3-OH, trihydroxylated anthocyanins; Met, methylated anthocyanins; Non-met, non-methylated anthocyanins. The asterisks denote p-values that indicate statistical significance (n = 40).*

## Discussion

Exogenous application of growth regulators is an important tool for improving the quality parameters of grape berries, including size, organoleptic characteristics, and color, among others (El-Kereamy et al., 2003; Han and Lee, 2004; Jeong et al., 2004; Symons et al., 2006; Fanzone et al., 2010; González et al., 2012; Xi et al., 2013). BRs compose a class of hormones involved in regulating berry ripening (Symons et al., 2006). However, the effects of exogenous applications of this type of growth regulator to table grapes have never been described. In fact, most studies on BRs as growth regulators have been performed on grapes for wine-make and involve the testing of only one BR analog, 24-epibrassinolide (Symons et al., 2006; Luan et al., 2013; Xi et al., 2013; Xu et al., 2015), thus limiting the understanding of the scope of this potential tool in a productive context in the case of table grapes.

In the current study, although no changes occurred in quality (total acidity, berry size) and yield (berry and cluster weight) parameters at harvest (Supplementary Tables 1, 2), a marked effect of BR analogs on soluble solids content, color of berries, concentration of anthocyanins, and the profiles of different groups of anthocyanins were observed (**Figures 2**–**4** and **Tables 3**, **4**). Compared with the control treatment, treatments E-0.4, B-2000, T-0.8, E-0.8, and T-0.4 were effective at increasing the soluble solids content of berries at harvest (**Figure 2**). Besides, results for treatments E-0.4 and B-2000 were consistent with those observed in the 2015–2016 season, in which these treatments were also statistically higher in soluble solids content than the control treatment. The rest of treatments, although showed average values higher than control treatment, did not present significant differences in soluble solids content. In addition, berries that received those treatments reached Brix values close to 15.5°, which is considerably higher than the 14.0° Brix values observed in berries that received the control treatment. Despite the absence of such differences prior to harvest, this finding suggests a faster increase of °Brix, which in a productive context means earlier harvest. This phenomenon is consistent with the reports by Luan et al. (2013) and Xi et al. (2013), who also observed an increase in the sugar content of berries as a consequence of 24-epibrassinolide applications. More recent studies have shown that this increase can be explained by the overexpression of hexose transporter genes *VvHT2*, *VvHT3*, *VvHT4*, *VvHT5*, and *VvHT6* as a consequence of 24-epibrassinolide application (Xu et al., 2015).

Brassinosteroids affect the accumulation of phenolic compounds, including anthocyanins (Symons et al., 2006; Xi et al., 2013); however, the exogenous application of BR analogs has not been previously demonstrated to be associated with the color of berries or with their evolution from veraison to harvest. In our study, compared with the control treatment, treatments E-0.4, T-0.4, T-0.8, and B-2000 effectively increased CIRG at harvest (**Figure 3A**). However, as noted above in the results, in season 2014–2015, the analysis of covariance revealed a significant effect of soluble solids on CIRG values. Despite these, when considering the effect of soluble solids content on CIRG, treatments E-0.4, T-0.8, and L-0.8 showed a significant direct effect in increasing CIRG, while the remains of the treatments did not show significant differences respect to control (**Figure 3B**). If the concentration and content of anthocyanins (**Figure 4**) are considered, the increase in color produced by BRs may occur on at least two levels: one level involves directly a higher levels of anthocyanins (**Figure 4**) and another level involving the effect of the sugar content (expressed as °Brix) on color (**Figure 2A**). In addition, the effects of BR applications on CIRG appear to depend on the analog and concentration used. For example, treatment T-0.4 (application of the Triol at 0.4 mg⋅L^-1^) appears to act by increasing the content of soluble solids, whereas treatment L-0.8 (application of the Lactone at 0.8 mg⋅L^-1^) appears to act by increasing the concentration of anthocyanins. Treatment E-0.4 (application of 24-epibrassinolide at 0.4 mg⋅L^-1^) and treatment T-0.8 (application of the Triol analog at 0.8 mg⋅L^-1^) appear to act on both levels. Although a significant effect of soluble solids content on CIRG values was not observed in the 2015–2016 season, the results for treatments E-0.4, T-0.4, and B-2000 (**Figure 3B**) are consistent with the observed in the previous season (**Figure 3A**), since CIRG values were also statistically higher than those of control treatment.

Aside from the effect of the soluble solids content on the color of the berries or growing season, berries of clusters that received BR treatments exhibited average CIRG values close to 3.9; according to Carreño et al. (1996), these values correspond to colors near red and pink (**Figure 5**), which are very attractive colors for “Redglobe” in the target markets.

Treatments E-0.4, T-0.4, L-0.8, and B-2000 and E-0.4, T-0.4, and B-2000 for seasons 2014–2015 and 2015–2016, respectively, yielded the highest total anthocyanin content and concentration relative to the control values (**Figure 4**). These results are in agreement with those of Symons et al. (2006), Luan et al. (2013), and Xi et al. (2013) regarding the role of this hormone in grape berry ripening, and they suggest a potential role for BR analog growth regulators as a table grape production management tool, especially considering the results obtained regarding berry coloration (**Figures 3**, **5**). In addition, despite that ANOVA results indicate only significant effects of the treatments on content and concentration of anthocyanins, a significant relationship was observed between soluble solids content and anthocyanins (data not shown). This type of effect has been described before, in that a higher sugar content is associated with greater synthesis and accumulation of anthocyanins (Hiratsuka et al., 2001).

The analysis of the effects of different concentrations of BR analogs (with the exception of the commercial formulation) revealed differences in CIRG values and total anthocyanin contents of berries. For example, when treatment E-0.4 (0.4 mg⋅L^-1^) was applied, a significant increase in CIRG values was observed relative to those of treatment E-0.8 (0.8 mg⋅L^-1^), which produced similar CIRG values to those of the control treatment (**Figures 3**, **4**). A similar behavior was observed in the case of the Triol analog, especially with respect to the effect on anthocyanin content: a concentration of 0.4 mg⋅L^-1^ (T-0.4) generated values that were significantly higher than in the control, whereas a concentration of 0.8 mg⋅L^-1^ (T-0.8) resulted in anthocyanin contents that were similar to those of the control. This type of dose–response relationship was already described by Luan et al. (2013) and Xi et al. (2013), who reported that concentrations of 24-epibrassinolide greater than 0.4 mg⋅L^-1^ produced the same levels of anthocyanins, sugars, and acidity in “Cabernet Sauvignon” plants as those found in untreated plants, suggesting the existence of an optimal concentration for stimulating the development of maturity. Interestingly, the Lactone analog generated a significantly higher CIRG values respect to control (**Figure 3B**) when applied at the highest concentration (L-0.8 treatment) rather than the lowest concentration (L-0.4 treatment). Compared with the control treatment, L-0.8 treatment produced a significantly greater total anthocyanin content, whereas L-0.4 treatment produced values similar to those of control. In this way, and unlike the results of the present study and others that involved 24-epibrassinolide (Luan et al., 2013; Xi et al., 2013), these findings suggest that Lactone analog applied at concentrations greater than 0.4 mg⋅L^-1^ might be the most effective for increasing CIRG and total anthocyanin content. The differences in the effects among analogs and their concentrations could be explained by differences in the affinity of the receptor or some of its components. In this sense, Clouse (2010) and Codreanu and Russinova (2011) reported that correct binding and/or interaction between BRs (natural or synthetic) and specific portions of the extracellular domain of the receptor is crucial for inducing a response.

A statistically significant correlation was observed between CIRG and total anthocyanin content (mg⋅berry^-1^) and concentration (mg⋅g FW^-1^) (**Table 5**). The results of a similar analysis but for different groups of anthocyanins revealed a significant correlation between the CIRG and 2-OH, Met and Non-met anthocyanins (**Table 5**). However, when anthocyanins are expressed as mg⋅g FW^-1^, the significance of the correlation extends to all 3-OH anthocyanins, with the exception of malvidin-3-glucoside (data not shown). This suggests that the CIRG values observed in this study (**Figures 3**, **5**) are associated with 2-OH anthocyanins. These results agree with those reported by Fernández-López et al. (1998), who associated the presence of 2-OH anthocyanins with grape varieties that have pink or red berries (CIRG ≈ 4.0). These authors also associated darker colors of berries (CIRG ≥ 6.5) with a greater abundance of 3-OH anthocyanins, particularly malvidin-3-glucoside.

Interestingly, three of the four treatments that resulted in significantly greater CIRG values than those from the control (E-0.4, T-0.4, and B-2000 in both seasons) also resulted in greater values of total anthocyanins and 2-OH anthocyanins, suggesting that the increase in total anthocyanin content occurred differentially toward this group of anthocyanins, i.e., those presenting pink and red colors. In fact, in berries of clusters that received these treatments, the 2-OH:3-OH anthocyanin ratios were close to 2.0 and 2.4 for the seasons 2014–2015 and 2015–2016, respectively (**Tables 3**, **4**). This differential accumulation is supported by the observed differences in the ratio between these two groups of anthocyanins (**Tables 3**, **4**). This phenomenon was particularly clear in berries that received treatments E-0.4 and B-2000 (in both seasons), which significantly differed from the control treatment in their 2-OH:3-OH ratio. The effect of the differential accumulation of anthocyanins could be explained by a varietal effect. As previously indicated, the predominant anthocyanins in “Redglobe” are of the 2-OH type (Carreño et al., 1997; Cantos et al., 2002; Peppi et al., 2007; Ustun et al., 2012), therefore an increase in the total anthocyanin concentration would mean an increase in the predominant type of anthocyanin present in the variety. However, a proportional increase in the amount of non-predominant anthocyanins could also occur. Nevertheless, this phenomenon was not observed in the present study, as indicated by the 2-OH:3-OH anthocyanin ratios (**Table 3**). Additionally, grape clusters treated with ABA exhibit increased total anthocyanin concentrations in berries, without altering the proportion of their different groups respect to the untreated berry clusters (Peppi et al., 2007). This is consistent with the idea that exogenous applications of BRs, especially treatments E-0.4 and B-2000, cause a differential increase in the amount of 2-OH anthocyanins present in berries. This increment could be caused by altered activity, expression, or post-transcriptional regulation of enzymes involved in the phenylpropanoid pathway, or in transcription factors controlling the expression of those enzymes, particularly flavonoid 3′-hydroxylase (F3’H), which is responsible for generating the precursor of cyanidin-3-glucoside (Bogs et al., 2005; Castellarin et al., 2006); methyltransferase (OMT), which is responsible for the methylation of cyanidin-3-glucoside, giving rise to peonidin-3-glucoside (Mattivi et al., 2006); and/or flavonoid 3′,5′-hydroxylase, that generates the precursor of delphinidin-3-glucoside (Bogs et al., 2005; Castellarin et al., 2006). Similar alterations in the expression and/or activity of the enzymes of the phenylpropanoid pathway occur in “Cabernet Sauvignon” as a consequence of high temperature (Mori et al., 2005), exposure to solar radiation (Matus et al., 2009), and even viral infection (Vega et al., 2011). Notwithstanding, no study has clearly indicated changes in the proportions of different groups of anthocyanins. So far, similar effects in table grapes have not been reported; thus, the present study is the first reporting changes of this type in table grapes and over two growing seasons. Nevertheless, additional studies are needed to determine how treatments E-0.4 and B-2000 give rise to this differential anthocyanin accumulation.

Although no comparisons between BRs and alternative methods for improving the berry color, such as the use of ethephon or ABA, were performed in the present study, the effect of BR treatments is attractive in a productive context given the types of colors they generate and the absence of detrimental effects on quality attributes of the treated clusters (**Figures 3**, **5**).

## Conclusion

The results of this study demonstrate that exogenous applications of different BRs analogs to “Redglobe” grape clusters result in a significant increase in berry color, in the soluble solids content, and total anthocyanins, altering the distribution of anthocyanin groups. This is the first report describing such effects on table grapes in a productive context.

The increase in the color of berries (expressed as CIRG) induced by treatment with BR analogs could be due to their effect on soluble solids content (treatments T-0.4 and B-2000), concentration of anthocyanins (treatment L-0.8), or the combination of these two effects in the berries (treatments E-0.4 and T-0.8).

The responses of CIRG and total anthocyanin content to the concentrations of the tested BR analogs show that Triols and 24-epibrassinolide have an effective concentration at approximately 0.4 mg⋅L^-1^, whereas Lactone appears to have a greater effect at concentrations higher than 0.4 mg⋅L^-1^.

This study showed that the activity of BR analogs is not restricted to just 24-epibrassinolide, which has been previously evaluated in other investigations, and that Triol, Lactone, and the commercial formulation (B-2000) were also active. The results demonstrate consistent effects of BRs on grape berries regardless of the analog used in two consecutive growing seasons.

Although several of the treatments performed in this study increased the total concentration of anthocyanins, only treatments E-0.4 and B-2000 resulted in a differential increase in 2-OH anthocyanins in two different growing seasons. This increment could chiefly explain the higher CIRG values observed in this work, which resulted in the expression of red and pink hues in berries, a color feature that is attractive for “Redglobe” markets.

Finally, this work demonstrates the potential of using BRs as a management tool in viticulture, and their use could at least complement the alternatives conventionally employed.

## Author Contributions

AV and AP-D designed the research. AV performed the research. AV, AP-D, and JA analyzed the data and results. LE, KD, and RC synthesized, characterized, and provided the two novel analogs brassinosteroid. AV wrote the paper.

## Conflict of Interest Statement

LE, KD, and RC declare conflict of interest because one of the analogous (Lactone) could be patented and/or protected by copyright. The other authors declare that the research was conducted in the absence of any commercial or financial relationships that could be construed as a potential conflict of interest.
