# Supplementary material for: Exogenous Applications of Brassinosteroids Improve Color of Red Table Grape (Vitis vinifera L. Cv. “Redglobe”) Berries
Source: Front Plant Sci. 2018 Apr 6;9:363. doi: 10.3389/fpls.2018.00363 (PMC5897653; doi:10.3389/fpls.2018.00363)
Supplement: Supplementary file 1 [file Table_1.docx]

Supplementary Material

Exogenous Applications Of Brassinosteroids Improve Color Of Red Table Grape (*Vitis vinifera* L. Cv. ‘Redglobe’) Berries

Alexis Vergara^1^, Katy Díaz^2^, Rodrigo Carvajal^2^, Luís Espinoza^2^, José A. Alcalde^1^, Alonso G. Pérez-Donoso^1*^

^1^Departamento de Fruticultura y Enología, Facultad de Agronomía e Ingeniería Forestal, Pontificia Universidad Católica de Chile, Santiago, Chile

^2^Departamento de Química, Universidad Técnica Federico Santa María, Valparaíso, Chile

*** Correspondence:**Alonso G. Pérez-Donoso
agperez@uc.cl

# Supplementary Figures and Tables

Supplementary Table 1. Effects of BR treatments on the diameter of berries, weight of berries, total acidity and weight of clusters at harvest for season 2014 – 2015. Each value indicates the mean of five replicates ± its standard deviation. n.s. = no statistical differences.

|  |  |  |  |  |  |  |  |  |
| --- | --- | --- | --- | --- | --- | --- | --- | --- |
| Treatments | Diameter | | Berry weight | | Total acidity | | Cluster weight | |
|  | (mm) | | (g) | | (g tartaric acid·L^-1^) | | (g) | |
| E-0.4 | 29.2 ± 1.20 | n.s | 17.0 ± 2.47 | n.s | 3.2 ± 0.28 | n.s | 1177.3 ± 265,05 | n.s |
| E-0.8 | 29.4 ± 1.11 | n.s | 18.0 ± 2.13 | n.s | 3.4 ± 0.26 | n.s | 1216.4 ± 192.27 | n.s |
| T-0.4 | 28.5 ± 2.01 | n.s | 15.3 ± 3.26 | n.s | 3.2 ± 0.64 | n.s | 1153.7 ± 183.56 | n.s |
| T-0.8 | 28.6 ± 1.11 | n.s | 15.9 ± 2.10 | n.s | 2.9 ± 0.24 | n.s | 1089.9 ± 209.53 | n.s |
| L-0.4 | 29.0 ± 1.19 | n.s | 16.6 ± 2.18 | n.s | 3.6 ± 0.30 | n.s | 1277.0 ± 267.08 | n.s |
| L-0.8 | 27.9 ± 0.99 | n.s | 15.5 ± 1.85 | n.s | 3.5 ± 0.38 | n.s | 1196.3 ± 138.79 | n.s |
| B-2000 | 28.7 ± 0.82 | n.s | 16.6 ± 1.75 | n.s | 3.4 ± 0.28 | n.s | 1282.6 ± 293.18 | n.s |
| Control | 28.5 ± 2.01 | n.s | 17.1 ± 2.06 | n.s | 3.4 ± 0.36 | n.s | 1169.1 ± 367.60 | n.s |
