# Supplementary material for: Exogenous Applications of Brassinosteroids Improve Color of Red Table Grape (Vitis vinifera L. Cv. “Redglobe”) Berries
Source: Front Plant Sci. 2018 Apr 6;9:363. doi: 10.3389/fpls.2018.00363 (PMC5897653; doi:10.3389/fpls.2018.00363)
Supplement: Supplementary file 2 [file Table_2.docx]

Supplementary Material

Exogenous Applications Of Brassinosteroids Improve Color Of Red Table Grape (*Vitis vinifera* L. Cv. ‘Redglobe’) Berries

Alexis Vergara^1^, Katy Díaz^2^, Rodrigo Carvajal^2^, Luís Espinoza^2^, José A. Alcalde^1^, Alonso G. Pérez-Donoso^1*^

^1^Departamento de Fruticultura y Enología, Facultad de Agronomía e Ingeniería Forestal, Pontificia Universidad Católica de Chile, Santiago, Chile

^2^Departamento de Química, Universidad Técnica Federico Santa María, Valparaíso, Chile

*** Correspondence:**Alonso G. Pérez-Donoso
agperez@uc.cl

# Supplementary Figures and Tables

Supplementary Table 2. Effects of BR treatments on the diameter of berries, weight of berries, total acidity and weight of clusters at harvest for season 2015 – 2016. Each value indicates the mean of five replicates ± its standard deviation. n.s. = no statistical differences.

|  |  |  |  |  |  |  |  |  |
| --- | --- | --- | --- | --- | --- | --- | --- | --- |
| Treatments | Diameter | | Berry weight | | Total acidity | | Cluster weight | |
|  | (mm) | | (g) | | (g tartaric acid·L^-1^) | | (g) | |
| E-0.4 | 27.2 ± 0.56 | n.s | 14.2 ± 0.68 | n.s | 3.4 ± 0.17 | n.s | 797.7 ± 143.69 | n.s |
| T-0.4 | 27.2 ± 1.09 | n.s | 14.3 ± 1.70 | n.s | 3.6 ± 0.21 | n.s | 795.3 ± 128.65 | n.s |
| L-0.4 | 27.5 ± 0.68 | n.s | 14.6 ± 0.72 | n.s | 3,4 ± 0.41 | n.s | 738.9 ± 73.63 | n.s |
| B-2000 | 28.0 ± 0.30 | n.s | 14.9 ± 0.70 | n.s | 3.4 ± 0.31 | n.s | 736.1 ± 63.69 | n.s |
| Control | 27.8 ± 0.81 | n.s | 14.1 ± 1.21 | n.s | 3.3 ± 0.16 | n.s | 749.5 ± 126.33 | n.s |
